# Gene therapy during *ex situ* heart perfusion: a new frontier in cardiac regenerative medicine?

**DOI:** 10.3389/fcvm.2023.1264449

**Published:** 2023-10-16

**Authors:** Mats T. Vervoorn, Jantijn J. G. J. Amelink, Elisa M. Ballan, Pieter A. Doevendans, Joost P. G. Sluijter, Mudit Mishra, Gerard J. J. Boink, Dawn E. Bowles, Niels P. van der Kaaij

**Affiliations:** ^1^ Division of Heart & Lungs, Department of Cardiothoracic Surgery, University Medical Center Utrecht, Utrecht, Netherlands; ^2^ Laboratory of Experimental Cardiology, Division Heart & Lungs, Department of Cardiology, University Medical Center Utrecht, Utrecht, Netherlands; ^3^ Netherlands Heart Institute, Utrecht, Netherlands; ^4^ Department of Cardiology, Division Heart & Lungs, University Medical Center Utrecht, Utrecht, Netherlands; ^5^ Regenerative Medicine Utrecht, Circulatory Health Research Center, University Utrecht, Utrecht, Netherlands; ^6^ Amsterdam Cardiovascular Sciences, Department of Medical Biology, Amsterdam University Medical Centers, Amsterdam, Netherlands; ^7^ Amsterdam Cardiovascular Sciences, Department of Cardiology, Amsterdam University Medical Centers, Amsterdam, Netherlands; ^8^ Divison of Surgical Sciences, Department of Surgery, Duke University School of Medicine, Durham, NC, United States

**Keywords:** gene therapy, heart transplantation (HTx), heart failure, regenerative medicine, *ex situ* heart perfusion

## Abstract

*Ex situ* organ preservation by machine perfusion can improve preservation of organs for transplantation. Furthermore, machine perfusion opens up the possibilities for selective immunomodulation, creation of tolerance to ischemia-reperfusion injury and/or correction of a pathogenic genetic defect. The application of gene modifying therapies to treat heart diseases caused by pathogenic mutations during *ex situ* heart perfusion seems promising, especially given the limitations related to delivery of vectors that were encountered during clinical trials using *in vivo* cardiac gene therapy. By isolating the heart in a metabolically and immunologically favorable environment and preventing off-target effects and dilution, it is possible to directly control factors that enhance the success rate of cardiac gene therapy. A literature search of PubMed and Embase databases was performed to identify all relevant studies regarding gene therapy during *ex situ* heart perfusion, aiming to highlight important lessons learned and discuss future clinical prospects of this promising approach.

## Introduction

With the introduction of machine perfusion for organ preservation, a new era within transplantation medicine has emerged. While conventional static cold storage is still the most commonly employed method for organ preservation, it is associated with a number of limitations, including tissue damage by prolonged hypothermia, limited possibilities for quality assessment, inevitable ischemia-reperfusion injury upon rewarming and reperfusion, and limited options for organ reconditioning. With the growing use of organs of marginal quality from extended criteria donors, these limitations impair clinical transplantation and contribute to the increasing supply-demand mismatch of donor organs (1, 2). By using machine perfusion, it is possible to overcome these limitations by providing a controlled flow of perfusate with a desired composition at a prespecified temperature, thereby facilitating the maintenance of tissue metabolism and removal of waste-products, while also serving as a platform for quality assessment and organ reconditioning (1).

In the context of the heart, the introduction of *ex situ* heart perfusion (ESHP) has increased the pool of available donors. It has done so by facilitating transplantation of hearts from extended-criteria donors, hearts from donors in remote geographical areas, and hearts donated after circulatory death, all with satisfactory clinical outcome (3–9). Following the increasing clinical use of ESHP, one can speculate about other uses for ESHP besides facilitating organ preservation, such as biological modification to improve clinical outcome (10–12). One such approach is cardiac gene therapy. In short, gene therapy can be defined as the delivery of therapeutic genetic material by different carriers (vectors) to cells with the aim of preventing or curing a disease by modification of a critical pathophysiological pathway or correction of a genetic defect (13). Most clinical *in vivo* cardiac gene therapy trials, however, yield overall unsatisfactory outcomes, to an important extent due to inadequate delivery and uptake of the viral vectors and expression of the gene product (14–17). These studies mostly used direct myocardial injection, or (percutaneous) intracoronary infusion as a means of vector delivery. In addition, concerns surrounding systemic side effects limit its applicability. This is perhaps best reflected by very high vector dose requirements for the most frequently used viral vector system, adeno-associated viral (AAV) vectors, that result in substantial (undesired) transduction of other organ systems such as the liver (18), leading to inflammatory stimulation (19) and potential activation of proto-oncogenes or disruption of tumor suppressor genes due to viral vector integration (13, 20–22).

**Figure 1:**
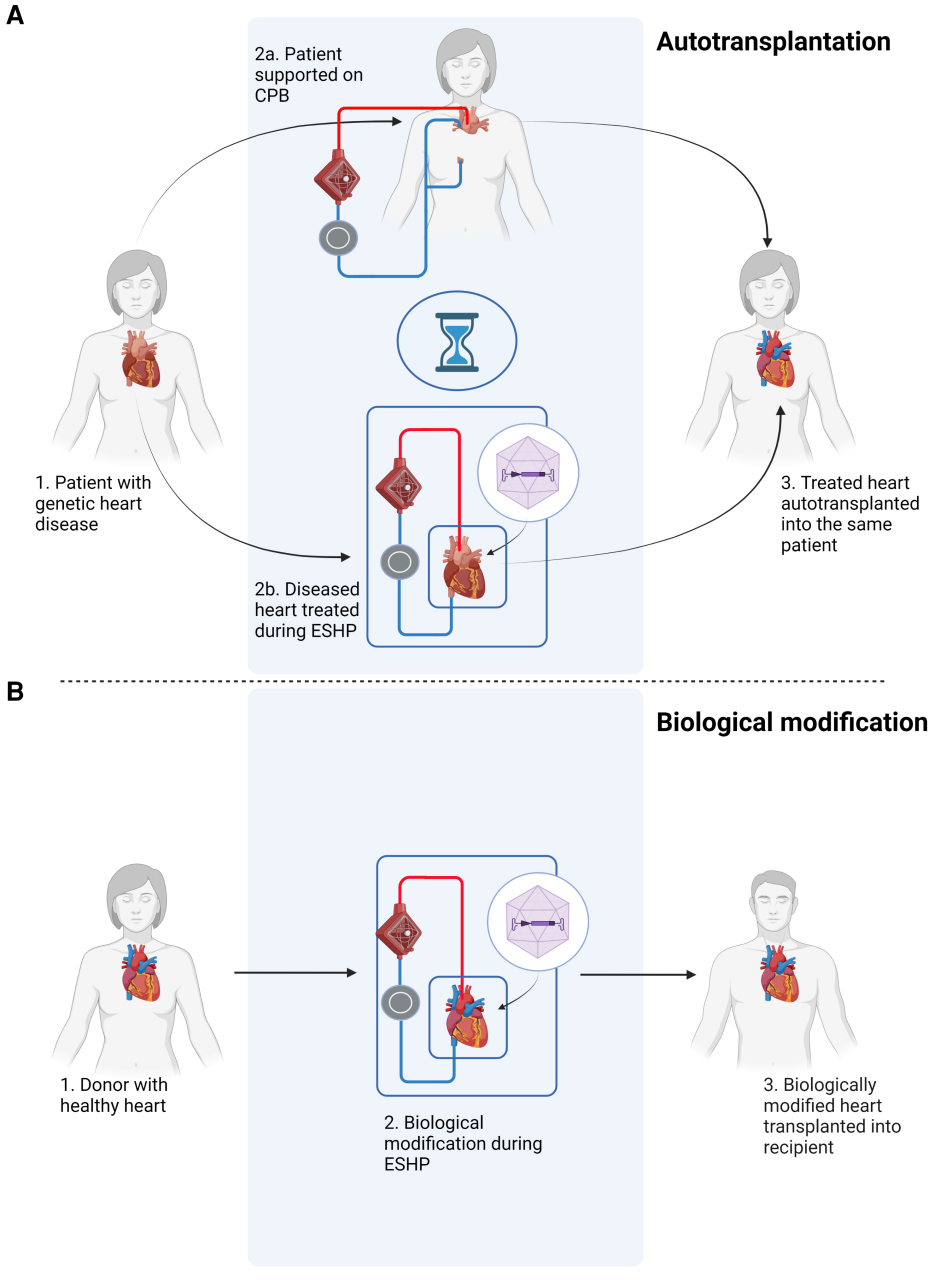
Schematic overview of potential applications of gene therapy during *ex situ* heart perfusion (ESHP). (**A**) Gene therapy of a heart with a pathogenic mutation, followed by autotransplantation. The patient is connected to a cardiopulmonary bypass (CPB) circuit during *ex situ* treatment of the diseased heart. (**B**) Gene therapy during *ex situ* heart perfusion for biological modification, e.g. immunomodulation, followed by orthotopic heart transplantation. The figure was constructed using Biorender.com.

ESHP provides the unique opportunity for local or intracoronary delivery of high concentrations of vectors without significant systemic off-target organ or immunological side effects. By isolating the heart in a metabolically favorable environment during ESHP, higher concentrations of vectors can be administered without systemic side effects. This opens up the possibility to modulate the inflammatory response associated with allotransplantation, thereby reducing the need for immunosuppression in recipients (23); improve tolerance to ischemia-reperfusion injury to reduce the risk of primary graft dysfunction; or excision and selective treatment of the heart to correct a pathogenic genetic defect at an early stage in a known carrier, followed by reimplantation of the heart into the same patient (autotransplantation) (Figure 1). The latter would prevent the need for heart transplantation in this subset of patients altogether. Given the fact that genetic causes play a substantial role in the etiology of many cases of heart failure (24–27), gene therapy during ESHP followed by autotransplantation might be especially relevant as a potential treatment option for carriers of known pathogenic mutation, preferably at earlier stages before a clinical phenotype has developed. Furthermore, interventions to treat these pathogenic mutations during ESHP could also be applied during isolated *in situ* loco-regional perfusion of the heart (28), which is currently being developed for clinical application [e.g., DiNAQOR AG (Schlieren, Switzerland)].

The objective of this systematic review is to summarize the available literature regarding the application of gene therapy during ESHP and discuss future clinical prospects based on the evidence found in the literature.

## Materials and methods

A literature search of the PubMed and Embase databases was conducted up to the 1st of July 2023. The search string is available in the appendix. Identified articles were uploaded to EndNote (Clarivate Analytics, Philadelphia, USA) for duplicate removal. After duplicate removal, the remaining studies were uploaded to Rayyan.ai for title/abstract screening. Studies were included if gene therapy was investigated during ESHP and ESHP was specifically used as a platform for intervention in small and large animal models. ESHP was defined as any form of coronary perfusion through the aortic root after excision of the heart, including bolus injection. Studies not adhering to this definition of ESHP (for instance selective intracoronary infusion or single flush after aortic cross clamping but before excision) were excluded. Remaining studies were subjected to full-text analysis before inclusion into our review. Reference lists of included articles were searched to identify additional studies. Title/abstract screening and full-text analysis was conducted by two authors independently (MTV, JJGJA). Disagreements were resolved by discussion and, if necessary, consultation of a third researcher not involved with the search and selection process (MM).

## Results

A total of 2,462 studies were identified after duplicate removal. After screening and full-text analysis, a total of 23 studies that specifically addressed the application of gene therapy during ESHP were retained. Based on the identified studies, we made a distinction between gene therapy applied during hypothermic ESHP (Table 1) and normothermic ESHP (Table 2), with hypothermic ESHP being the focus of most papers.

**Table 1:** Studies that utilize hypothermic *ex situ* heart perfusion.

Author	Year	Animal model	Used Vector	Used Gene	Perfusion setting	Results
Adenoviral vectors						
Shiraishi et al. (29)	1996	Rat	Adenovirus (AdCMVLacZ)	LacZ	20 ml of virus-containing UW solution at 4°C for 20 min at a flow of 1 ml/min with different titers and total preservation time, followed by heterotopic transplantation.	• Infection and gene delivery had no adverse effect on graft survival. • Gene expression returned to baseline after 14 days.
Gojo al. (30)	1998	Rat	Adenovirus (Adex1CALacZ)	LacZ	50 ml of virus-containing UW solution at 4°C for 60 min with different titers and total preservation time, followed by heterotopic transplantation.	• Infection and gene delivery had no adverse effect on graft survival. • Gene expression universally returned to baseline after 5 weeks. • Increased leukocyte infiltration with titers >1 × 10 (10) PFU/ml, with earlier reduction in gene expression
Abunasra et al. (31)	2003	Rat	Adenovirus (AdCMVLacZ, AdeNOS & AdMnSOD)	LacZ, eNOS, Mn-SOD	5 ml of virus-containing UW solution at 4°C for 15 min at a flow of 0.75 ml/min with different titers, followed by heterotopic transplantation.	• Infection and gene delivery had no adverse effect on contractile function. • Improved recovery of contractile functions after induced ischemia-reperfusion insult in the eNOS and Mn-SOD groups.
Yap et al. (32)	2001	Rat	Adenovirus (AdCMVLacZ)	LacZ	Virus-containing 2% fetal calf serum at 4°C for 5 s with different titers into the aortic root, followed by 60 min of storage and heterotopic transplantation.	• Increased infection and gene delivery with longer exposure times • Increased infection and gene delivery with higher titers [up to 1. × 10 (10) PFU/ml]. • Preferred gene expression in cardiomyocytes over other cell types. • Accentuated expression in areas of ischemia. • No inflammatory cell infiltration after 4 days.
Pellegrini et al. (33)	1998	Rat	Adenovirus (AdCMVLacZ)	LacZ	Virus-containing [1 × 10 (9) PFU/ml] 2% fetal calf serum at 4°C for 5 s into the aortic root, followed by 60 min of storage and heterotopic transplantation.	• Preferred gene expression in cardiomyocytes over other cell types. • Accentuated expression in areas of ischemia. • No inflammatory cell infiltration after 1 week.
Brauner et al. (34)	1997	Rabbit	Adenovirus (AdSvIL10 & AdCMVTGF-*β*1)	IL10, TGF-beta 1	20 ml of virus-containing UW solution at 4°C for 15 min at a flow of 1 and 0.5 ml/min, followed by heterotopic transplantation.	• Increased infection and gene delivery with continuous perfusion compared to bolus injection. • Significant IL10 and TGF-beta 1 expression in infected hearts, that increased with higher titers.
Brauner et al. (35)	1997	Rabbit	Adenovirus (AdSvIL10 & AdCMVTGF-β1)	IL10, TGF-beta 1	20 ml of virus-containing UW solution at 4°C for 15 min at a flow of 1 and 0.5 ml/min, followed by heterotopic transplantation.	• Myocardial distribution improved after increasing perfusion pressure and adding pulsatility. • Acute allograft rejection was decreased after cytokine gene therapy.
Yang et al. (36)	1999	Rat	Adenovirus (AdCTLA4Ig & AdLacZ)	LacZ, CTLA4Ig	1 ml of virus-containing [1 × 10 (11) PFU/ml] saline solution at 4°C for 10–15 min followed by heterotopic transplantation in in non-immunosuppressed mismatched rats.	• AdCTLA4Ig-treated hearts demonstrated indefinite survival in non-immunosuppressed mismatched recipients. • No lymphocytic cell infiltration was noted in the AdCTLA4Ig-treated hearts. • Gene expression was abundant in the endo-myocardium • Gene expression was not detected after 100 days.
Pellegrini et al. (37)	2000	Rat	Adenovirus (AdCMVLacZ)	LacZ	5 ml of virus-containing UW solution at 4°C for varying infection intervals with different flows and pressures, followed by heterotopic transplantation.	• Increased infection and gene delivery with continuous perfusion compared to bolus injection. • Preferred gene expression in cardiomyocytes over other cell types. • Higher titers were associated with higher levels of inflammatory infiltration. • Perfusion pressures >50 mmHg were associated with more tissue damage.
Oi et al. (38)	2006	Porcine	Adenovirus (AdCMVLacZ)	LacZ	200 ml of virus containing UW solution at 4°C with varying titers, for 30 min with a perfusion pressure of 50 mmHg, followed by heterotopic heart transplantation.	• Increased infection and gene delivery with higher titers [up to 1 × 10 (9) PFU/ml]. • Homogeneous distribution across the myocardium was achieved. • Virus titers did not correlate with edema formation.
Rao et al. (39)	2007	Rat	Adenovirus (AdCMVLacZ)	LacZ	5 ml of virus-containing [3.5 × 10 (8) PFU] UW solution at 4°C for 15 min at a flow of 0.75 ml/min with different titers, followed by heterotopic transplantation.	• Infection and gene delivery had no adverse effect cardiac allograft vasculopathy development.
Abunasra et al. (40)	2006	Rat	Adenovirus (AdCMVLacZ & AdMnSOD)	LacZ, Mn-SOD	5 ml of virus-containing UW solution at 4°C for 15 min at a flow of 0.75 ml/min with different titers, followed by heterotopic transplantation.	• Increased infection and gene delivery with continuous perfusion compared to bolus injection. • Infection and gene delivery had no adverse effect on contractile function. • Improved recovery of contractile functions after induced ischemia-reperfusion insult in the Mn-SOD group.
Ricci et al. (41)	2010	Rat	Adenovirus (AdCMVhNIS)	NIS	5 ml of virus-containing [1 × 10 (9) PFU/ml] UW solution at 4°C, followed by heterotopic transplantation and injection of ^131^I after 3 days.	• Successful gene transfer of the NIS-gene could be confirmed. • Graft survival was significantly higher in AdCMVhNIS-treated hearts following injection of ^131^I. • No inflammatory infiltrates were found in AdCMVhNIS-treated hearts following injection of ^131^I.
Adeno-associated viral vectors						
Miyagi et al. (42)	2008	Rat	Adeno-associated virus (rAAV9CMVLacZ)	LacZ	5 ml of virus-containing UW solution at 4°C with different titers for 20 min at a flow of 0.75 ml/min, followed by heterotopic transplantation and injection of ^131^I after 3 days.	• Increased infection and gene delivery with higher titers (up to 2 × 10 (12 vector genomes/ml). • There was no difference in gene expression between ESHP perfusion and intravenous injection. • Durable and stable gene transfer was achieved for 3 months.
Liposome-based vectors						
Furukawa et al. (43)	2005	Rabbit	Liposome (pSVhIL-4, pSVhIL-10	IL4, IL10	10 ml of liposome-containing saline solution at 4°C for 30 min with a flow of 20 ml/min, followed by heterotopic transplantation in mismatched recipients	• Successful gene transfer could be confirmed. • Expression reached a peak at 7–8 days, followed by a slow decline. • Increased infection and gene delivery with higher titers. • No systemic wash-out was noted in recipients. • Preferred gene expression in cardiomyocytes over other cell types. • Mean allograft survival was significantly prolonged from 9 to 135 days. • There was a synergistic effect on allograft survival when both genes were delivered, potentially due to suppression of T lymphocyte infiltration induced by localized overexpression of Il4 and IL10.
Jayakumar et al. (44)	2000	Rat	Liposome (HVJ-liposome containing HSP70 DNA)	HSP70	1 ml of liposome-containing fluid through the aortic root, followed by heterotopic transplantation and subsequent excision after 4 days for ischemia-reperfusion challenge during Langendorff perfusion.	• Successful gene transfer could be confirmed. • Improved recovery of contractile functions after induced ischemia-reperfusion insult in the HSP70 group. • Significantly higher recovery of endothelial function after induced ischemia-reperfusion insult in the HSP70 group.

UW, University of Wisconsin; PFU, plaque-forming units; eNOS, endothelial nitric oxide synthase; Mn-SOD, manganese superoxide dismutase; NIS: sodium-iodide symporter; HVJ, Hemagglutinating Virus of Japan; HSP, heat-shock protein.

**Table 2:** Studies that utilize normothermic *ex situ* heart perfusion.

Author	Year	Animal model	Used Vector	Used Gene	Perfusion setting	Results
Adenoviral vector						
Donahue et al. (45)	1997	Rabbit	Adenovirus (AdCMVLacZ)	LacZ	50 ml of virus-containing oxygenated KHB at 37°C, flows between 10 and 40 ml/min, pressures between 10 and 70 mmHg, varying infection intervals up to 180 min.	• Increased infection and gene delivery at flows >30 ml/min. • Increased infection and gene delivery with longer exposure times. • Increased infection and gene delivery with higher virus titers [up to 1.6 × 10 (9) PFU/ml].
Donahue et al. (46)	1998	Rabbit	Adenovirus (AdCMVLacZ)	LacZ	Virus-containing oxygenated KHB at 37°C with varying flows, pressures and infection intervals, for a total Langendorff time of 180 min. Pretreatment with calcium-KHB, KHB supplemented with bradykinin, serotonin, L-NAME, heparinized blood.	• Increased infection and gene delivery with longer exposure times. • Increased infection and gene delivery with higher titers [up to 1.6 × 10 (9) PFU/ml]. • Increased infection and gene delivery after pretreatment with agents that increase microvascular permeability. • Synergistic effect after combination of the above mentioned factors.
Nagata et al. (47)	2001	Rabbit	Adenovirus (AdCMVLacZ)	LacZ	Virus-containing [1 × 10 (8)PFU/ml oxygenated KHB at 37°C. Infection interval was fixed at 2 min, for a total Langendorff time of 180 min. Pretreatment with VEGF, TNG, 8Br-cGMP, L-NMMA, ODQ, sildenafil, zaprinast.	• Increased infection and gene delivery after pretreatment with agents that increase available nitric oxide. • Synergistic effect after combination of agents that increase available nitric oxide.
Lehnart et al. (48)	2000	Rabbit	Adenovirus (AdCMVLacZ & AdRSVLuc)	LacZ & Luciferase	25 ml of virus-containing [1.6 × 10 (9) PFU/ml] oxygenated KHB at 37°C recirculated for 60 min at a flow of 30 ml/min.	• No adverse effect on contractile and diastolic properties over 48 h of functional evaluation. • Infection and gene delivery had no adverse effect on response to beta-adrenergic stimulation.
Bishawi et al. (49)	2019	Porcine	Adenovirus (AdCMVLuc)	Luciferase	Virus-containing [5 × 10 (13) PFU/ml] solution mixed with crystalloid prime and washed RBC's, perfusion for 2 h at a flow of 600 ml/min, perfusion pressure of 65–70 mmHg, followed by flushing and heterotopic transplantation.	• Complete inhibition of infection and gene delivery by plasma and serum. This effect was minimized by using washed RBC's in combination with crystalloid prime. • Successful infection and gene delivery across all areas of the heart, as well as the coronary arteries. • Accentuated infection and gene delivery in right ventricular and septal areas when compared to the left ventricle. • No systemic wash-out after flushing and heterotopic transplantation
Adeno-associated viral vectors						
Mendiola Pla et al. (50)	2023	Porcine	Adeno-associated virus (AAV3b SASTG)	Luciferase	Virus-containing solution with different titers mixed with crystalloid prime and washed RBC's, perfusion for 2 h at a flow of 600 ml/min, perfusion pressure of 60–70 mmHg, followed by flushing and heterotopic transplantation.	• High transduction efficiency of SASTG • Most uptake (83.82%) within the first 30 min • Solid transgene expression up to 35 days without off-target effects or signs of rejection • Dose-dependent effect.

KHB, krebs-henseleit buffer; PFU, plaque-forming units; L-NAME, N(*ω*)-nitro-L-arginine methyl ester; VEGF, vascular endothelial growth factor; TNG, trinitroglycerin; 8Br-cGMP, 8-Bromoguanosine 3′,5′-cyclic monophosphate; L-NMMA, N^G^-monomethyl-L-arginine; ODQ, Oxadiazolo-[4,3-a]-quinoxalin-1-one.

### Gene therapy during hypothermic *ex situ* heart perfusion

Most research into gene therapy during hypothermic ESHP involved adenoviral (Ad) mediated gene transfer (29–41). Three studies could be identified that involved AAV (42) or liposome-mediated gene transfer (43, 44). Hearts were mostly harvested from rats (29–33, 36, 37, 39–42, 44) or rabbits (34, 35, 43), one study involved porcine hearts (38).

#### Perfusion conditions

The identified studies universally defined hypothermic machine perfusion as perfusion of the cardiac vasculature at ±4°C. The perfusate solution consisted of acellular University of Wisconsin solution (29–31, 34, 35, 37–42), saline (36, 43) or fetal calf serum (32), and reported perfusion duration varied between 5 s (*n* = 2 studies), 15 min (*n* = 6 studies), 20 min (*n* = 2 studies), 30 min (*n* = 2 studies) and 60 min (*n* = 3 studies), with most studies using perfusion duration of 15–30 min.

When comparing continuous perfusion to a single bolus injection, multiple studies have demonstrated that transduction efficiency is superior with continuous perfusion compared to single bolus injection (34, 35, 37, 40). Evidence suggests that administration of the vector with a pulsatile perfusion pressure might further improve efficiency and transmyocardial distribution of the infused vector (35). Furthermore, perfusion pressure seems to be inversely associated with the required perfusion duration for satisfactory transduction, i.e., an increase in perfusion pressure results in a reduction in the time needed to achieve the same level of transduction (35, 37). It must be noted, however, that higher perfusion pressures (70–80 mmHg) are associated with increased histological tissue damage and edema formation compared to lower perfusion pressures (<50 mmHg) during hypothermic ESHP, and might damage the graft (37). Regarding edema formation, one study mentioned that Ad vector titers up to 1 × 10(9) plaque-forming units (PFU)/ml did not correlate with increased edema formation. Suggesting that any edema formation observed at titers lower than 1 × 10(9) PFU is most likely the result of the perfusion itself (38).

Based on the available literature, we can assume that there is a dose-dependent effect for Ad mediated gene therapy, with improved transduction at higher viral titers. However, due to heterogeneity among studies regarding specific titer definition (i.e., PFU as single vector dose, per unit perfusate volume or unit of heart weight) it is difficult to define the optimal titer range on the basis of prior literature. Furthermore, optimal titer range might also be dependent on the specific subtype of Ad (32, 34, 38). However, too high doses might result in microcirculatory obstruction [reported single vector dose ≥1 × 10(11)] (29) and an increased inflammatory response in the myocardium [reported single vector dose ≥1 × 10(10)] (30), resulting in poor myocardial function and a reduced length of gene expression (≥4 weeks with lower doses vs. 3 weeks with higher doses). The inflammatory response associated with too high doses consisted of increased leukocyte infiltration surrounding transducted cells distributed across the myocardium up to 21 days after heterotopic transplantation.(30) These findings also suggest that the level of inflammation can negatively impact duration of expression, and thus effectivity, of gene therapy during ESHP. A positive dose-dependent relationship has also been reported in liposome mediated delivery of genes (43) and with the use of AAV-based vectors (42).

In summary, these results suggest that during hypothermic ESHP continuous, pulsatile perfusion pressures with a mean pressure of 50 mmHg seem optimal. The most appropriate titer for optimal transduction is up to debate, but seems to follow a parabolic trend based on evidence that suggests a dose-dependent effect below a certain threshold, followed by a range of optimal transduction and eventually a point where higher titers are associated with graft dysfunction and reduced efficacy of transduction and expression.

#### Myocardial distribution

Reported myocardial distribution of transgene expression is heterogeneous and studies mainly report perfusion pressure and pulsatility as factors of influence. The effects of perfusion solution, reperfusion strategy, position of the heart and antegrade vs. retrograde perfusion on myocardial distribution was not reported. Among studies using Ad vectors, both Gojo (30), Pellegrini (33, 37) and Yap (32) reported preferred expression in cardiomyocytes over endothelial cells, while Brauner et al. (34) reported high expression in subepicardial perivascular regions (100% transduction rate) and lower in mid-wall and subendocardial regions (5%–20%). This difference in expression pattern among regions, however, was reduced by increasing perfusion pressure and adding pulsatility, resulting in a more equal distribution (25%–40%) across the myocardium. On the contrary, Yang et al. (36) report abundant expression in the endomyocardial tissue, but lower in the mid layers of myocardium using an antegrade perfusion approach. They did not report on specific perfusion pressure to explain these differences in distribution. Pellegrini et al. (33, 37) also report accentuated transgene expression in the right ventricle, especially in the subepicardial region, upon infusion of 1 × 10(9) PFU/ml Ad vector at different perfusion pressures (up to 40–50 mmHg) and exposure times. They also noted increased expression around zones that endured (warm) ischemia, possibly due to locally increased endothelial permeability associated with (warm) ischemia (33). Oi et al. (38) report a homogeneous distribution of expression across a multitude of left ventricular segments after using a continuous perfusion pressure of 50 mmHg, suggesting that heterogeneous distribution is less of a concern when higher perfusion pressures are used. In models using liposome-mediated transfer, one study reported homogeneously distributed transfection after 30 min of cold perfusion at a non-specified perfusion pressure (43). No reports on myocardial distribution were identified using AAV.

Taken together, these results suggest that pulsatile perfusion pressures centered around a mean pressure of 50 mmHg seems optimal to achieve a homogeneous distribution during hypothermic ESHP.

#### Therapeutic interventions

**Figure 2:**
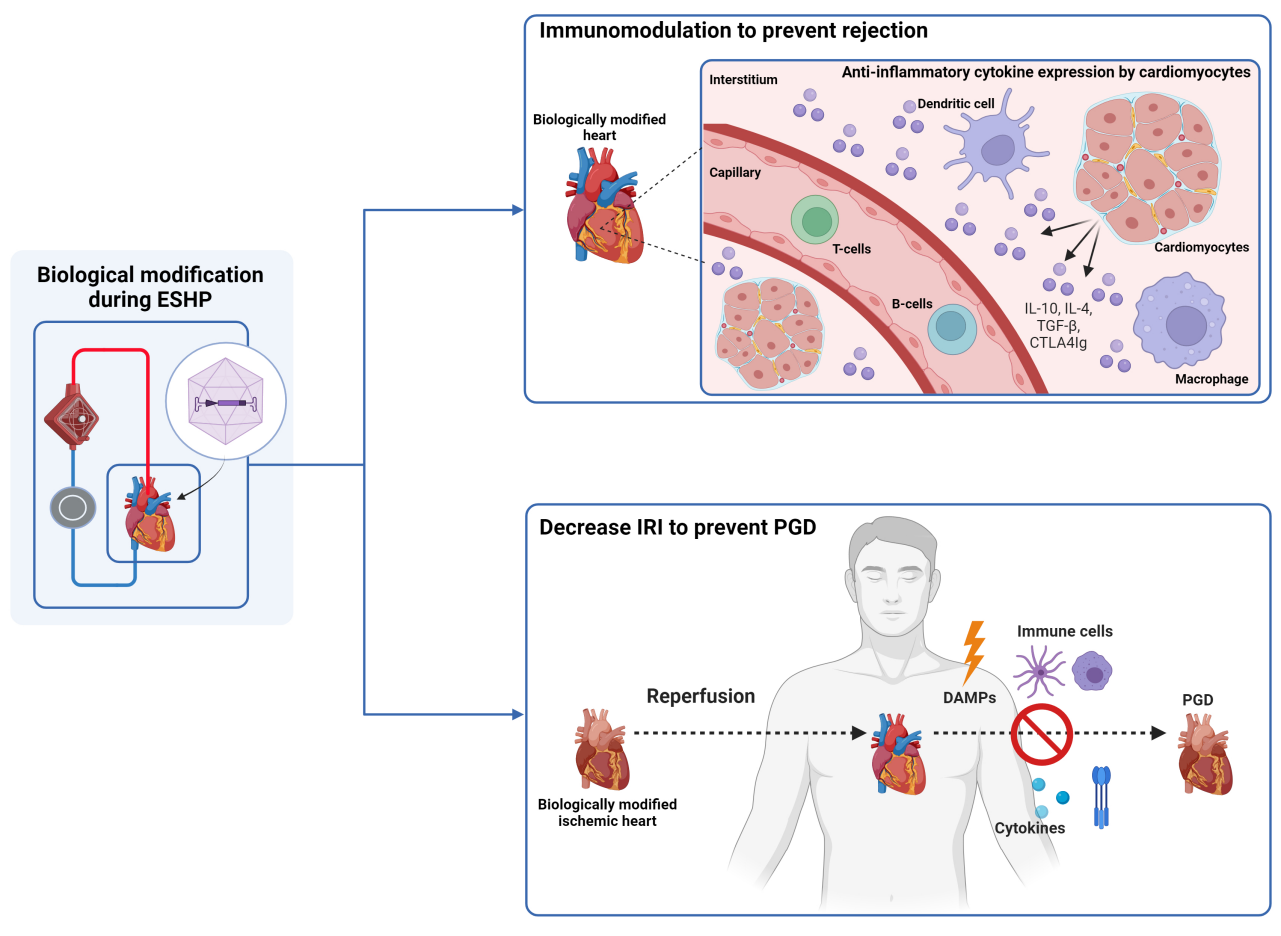
Gene therapy during ESHP has been investigated for immunomodulation and to decrease ischemia-reperfusion injury (IRI). Immunomodulation was conducted by transduction of cardiomyocytes with adenoviral vectors that carried genes encoding anti-inflammatory cytokines (IL-4, IL-10, TGF-beta, and CTLA4IG. IRI was attenuated by adenoviral mediated gene transfer of manganese superoxide dismutase, nitric oxide synthase and heat shock protein 70. ESHP: ex situ heart perfusion. IL: interleukine. PGD: primary graft dysfunction. DAMPS: damage-associated molecular pattern. The figure was constructed using Biorender.com.

Regarding therapeutic potential, some studies assessed the efficacy of gene therapy during ESHP for immunomodulation, or to increase tolerance of the graft to ischemia-reperfusion injury (Table 3; Figure 2). One study assessed the influence of Ad vectors on the development of cardiac allograft vasculopathy.

**Table 3:** Studies that investigated the therapeutic potential of gene therapy during hypothermic ESHP.

Author	Year	Animal model	Used Vector	Used Gene	Perfusion setting	Results
Adenoviral vectors						
Brauner et al. (34)	1997	Rabbit	Adenovirus (AdSvIL10 & AdCMVTGF-β1)	IL10, TGF-beta 1	20 ml of virus-containing UW solution at 4°C for 15 min at a flow of 1 and 0.5 ml/min, followed by heterotopic transplantation.	• Significant IL10 and TGF-beta 1 expression in infected hearts, that increased with higher titers.
Brauner et al. (35)	1997	Rabbit	Adenovirus (AdSvIL10 & AdCMVTGF-β1)	IL10, TGF-beta 1	20 ml of virus-containing UW solution at 4°C for 15 min at a flow of 1 and 0.5 ml/min, followed by heterotopic transplantation.	• Acute allograft rejection was decreased after cytokine gene therapy.
Yang et al. (36)	1999	Rat	Adenovirus (AdCTLA4Ig & AdLacZ)	LacZ, CTLA4Ig	1 ml of virus-containing [1 × 10 (11) PFU/ml] saline solution at 4°C for 10–15 min followed by heterotopic transplantation in in non-immunosuppressed mismatched rats.	• AdCTLA4Ig-treated hearts demonstrated indefinite survival in non-immunosuppressed mismatched recipients. • No lymphocytic cell infiltration was noted in the AdCTLA4Ig-treated hearts.
Abunasra et al. (31)	2003	Rat	Adenovirus (AdCMVLacZ, AdeNOS & AdMnSOD)	LacZ, eNOS, Mn-SOD	5 ml of virus-containing UW solution at 4°C for 15 min at a flow of 0.75 ml/min with different titers, followed by heterotopic transplantation.	• Improved recovery of contractile functions after induced ischemia-reperfusion insult in the eNOS and Mn-SOD groups.
Abunasra et al. (40)	2006	Rat	Adenovirus (AdCMVLacZ & AdMnSOD)	LacZ, Mn-SOD	5 ml of virus-containing UW solution at 4°C for 15 min at a flow of 0.75 ml/min with different titers, followed by heterotopic transplantation.	• Infection and gene delivery had no adverse effect on contractile function. • Improved recovery of contractile functions after induced ischemia-reperfusion insult in the Mn-SOD group.
Ricci et al. (41)	2010	Rat	Adenovirus (AdCMVhNIS)	NIS	5 ml of virus-containing [1 × 10 (9) PFU/ml] UW solution at 4°C, followed by heterotopic transplantation and injection of ^131^I after 3 days.	• Graft survival was significantly higher in AdCMVhNIS-treated hearts following injection of ^131^I. • No inflammatory infiltrates were found in AdCMVhNIS-treated hearts following injection of ^131^I.
Liposome-based vectors						
Jayakumar et al. (44)	2000	Rat	Liposome (HVJ-liposome containing HSP70 DNA)	HSP70	1 ml of liposome-containing fluid through the aortic root, followed by heterotopic transplantation and subsequent excision after 4 days for ischemia-reperfusion challenge during Langendorff perfusion.	• Improved recovery of contractile functions after induced ischemia-reperfusion insult in the HSP70 group. • Significantly higher recovery of endothelial function after induced ischemia-reperfusion insult in the HSP70 group.
Furukawa et al. (43)	2005	Rabbit	Liposome (pSVhIL-4, pSVhIL-10	IL4, IL10	10 ml of liposome-containing saline solution at 4°C for 30 min with a flow of 20 ml/min, followed by heterotopic transplantation in mismatched recipients	• Mean allograft survival was significantly prolonged from 9 to 135 days. • There was a synergistic effect on allograft survival when both genes were delivered, potentially due to suppression of T lymphocyte infiltration induced by localized overexpression of IL4 and IL10.

UW, University of Wisconsin; PFU, plaque-forming units; eNOS, endothelial nitric oxide synthase; Mn-SOD, manganese superoxide dismutase; NIS, sodium-iodide symporter; HVJ, Hemagglutinating Virus of Japan; HSP, heat-shock protein.

A total of 5 studies have shown that it is possible to modulate the immune response associated with allograft implantation. Brauner et al. (34) constructed Ad vectors expressing viral interleukin-10 (AdSvIL10) or transforming growth factor-beta 1 (AdCMVTGF-beta 1), two anti-inflammatory cytokines, and delivered them to rabbit hearts. Following ESHP, the hearts were heterotopically transplanted in recipient rabbits. They reported successful expression of TGF-beta 1 and IL10 in the grafts, especially with higher vector concentrations, and no evidence of neoplastic side-effects, such as intimal proliferation, or pro-fibrotic effects, assessed after 4 days of follow-up. In a subsequent study (35), using the same model, the authors showed a significant inhibitory action on acute allograft rejection through immunosuppressive cytokine gene delivery, prolonging graft survival. Building on this experience, Yang et al. (36) used Ad vectors (AdCTLA4Ig) to deliver the CTLA4Ig gene to promote survival of grafts in a heterotopic transplantation model of Lewis and Brown Norway rat hearts. They reported abundant cardiac expression of the CTLA4Ig transgene after transplantation in non-immunosuppressed Wistar Furth recipients. Although progressive diminution of CTLA4Ig mRNA expression was noted over time, the allografts survived indefinitely with a sufficient degree of localized immunosuppression. Ricci et al. (41) studied the effects of gene transfer of human iodide symporter and subsequent treatment with ^131^I on acute allograft rejection using Ad vectors in a heterotopic rat transplantation model. They demonstrated significantly longer survival and reduced myocardial damage in grafts perfused with the Ad vectors containing the gene that received treatment with ^131^I. Finally, Furukawa et al. (43) used a liposomal-mediated approach to deliver a combination of IL4 and IL10 in a heterotopic transplantation model of rabbit hearts. They reported successful gene transfer and were able to prolong allograft survival in hearts that received both interleukins (9 ± 2 days vs. 135 ± 3 days) with a great improvement of histological rejection grades, indicating a synergistic action of both cytokines. This was presumably due to reduced alloreactivity of T-lymphocytes induced by localized overexpression of the interleukins. By reducing this alloreactivity, long-term survival of cardiac allografts without systemic immunosuppression was possible. Taken together, these results indicate that (local) immunomodulation of transplanted grafts is possible during ESHP, which might enable a less restrictive immunosuppressive regimen to prevent rejection in recipients.

A total of three studies demonstrated that the authors were able to enhance myocardial tolerance to ischemia-reperfusion injury, preserving both contractile and endothelial function of treated grafts. Abunasra et al. (31, 40) used a heterotopic transplantation model to demonstrate the protective effects of Ad mediated gene transfer of manganese superoxide dismutase and nitric oxide synthase into rat hearts. After heterotopic transplantation, the hearts were procured and reperfused on a Langendorff system for assessment and induction of an ischemic insult. After demonstrating successful gene transfer and expression, they noted improved recovery of contractile function after the ischemic insult in treated hearts. Jayakumar et al. (44) used a similar model to assess the effects of heat shock protein 70 (Hsp70) gene transfection on rat hearts using liposomal vectors for delivery. They demonstrated improved postischemic recovery of contractile function and recovery of coronary flow, together with reduced creatinine kinase release in hearts that were treated with Hsp70. These results indicate that myocardial ischemia-reperfusion injury can potentially be attenuated using gene therapy.

Finally, Rao et al. (39) used a heterotopic heart transplantation model to study the effects of Ad mediated gene transfer on the later development of cardiac allograft vasculopathy due to concerns over the potential effects of adenoviral therapy on the development of allograft vasculopathy. They concluded that Ad mediated gene transfer did not result in accelerated allograft vasculopathy development compared to non-treated controls after 120 days.

### Gene therapy during normothermic *ex situ* heart perfusion

Gene therapy research during normothermic ESHP mostly involved Ad mediated gene transfer (45–49) using rabbit (45–48) or porcine hearts (49). One study involved AAV mediated gene transfer in porcine hearts (50). The identified studies were notably lower in number compared to studies that assessed gene therapy during hypothermic ESHP (6 vs. 17).

Donahue et al. (45, 46) studied the optimal conditions for delivery of gene products during normothermic ESHP using Ad vectors in rabbit hearts. They found that transduction increased incrementally with coronary flow (up to 40 ml/min), exposure time (up to 120 min) and administered viral dose [up to 1.6 × 10(9) PFU/ml] (45). They also report further improvements in efficacy by minimizing calcium concentration in the perfusate and implementing pretreatment of the hearts with several pharmacological agents that increase microvascular permeability, such as bradykinin, serotonin and L-NAME. As an example they noted a transduction rate of over 90% in just two minutes of perfusion by combining hypocalcemia and serotonin administration, confirming the hypothesis that required exposure time could dramatically be reduced by modulating viral dose and vascular permeability (46). In a follow-up study using the same model, Nagata et al. (47) report that pretreatment with vascular endothelial growth factor combined with a phosphodiesterase inhibitor, such as nitroglycerin or sildenafil, could also increase the efficiency of gene transfer in a dose-dependent fashion. Furthermore, Lehnart et al. (48) demonstrated that transduction of cardiomyocytes with Ad vectors does not adversely affect contractile function of perfused hearts, strengthening the belief that gene therapy using Ad vectors has potential as a safe modality for therapeutic intervention. Further proof-of-concept was provided by Bishawi et al. (49), who demonstrated safe and efficacious Ad mediated gene transfer in a porcine heterotopic transplantation model. In this study, the authors used 2 h of normothermic ESHP as a platform to deliver 5 × 10(13) total viral particles of an Ad luciferase vector (AdCMVLuc) prior to allograft implantation in a blood type compatible recipient pig. Enzymatic assessment of luciferase activity obtained 5 days after transplantation revealed global and uniform luciferase activity in the allograft and coronary arteries, without systemic off-target expression. Interesting lessons learned during these experiments include the apparent inhibitory influences of plasma and serum on transduction efficiency. However, these inhibitory effects could be minimized when cell-salvaged erythrocytes were mixed with the priming solution instead of whole blood, although some inhibitory effects on transduction efficiency remained. Nevertheless, by using a perfusate solution based on crystalloid prime and cell-salvaged erythrocytes, they reported satisfactory expression across all areas of the heart after heterotopic transplantation into recipient pigs. Based on these findings, the authors conducted a subsequent study into the efficacy of AAV based delivery and transduction during ESHP using a similar model (50). Their most important finding was that they were able to achieve durable transgene expression using AAV-mediated gene transfer for up to 35 days following heterotopic transplantation, without signs of systemic off-target expression, rejection or inflammation in the graft. Furthermore, they identified SASTG, a myocardial-enhanced AAV3b variant, as the most efficient vector to deliver transgenes when used during normothermic ESHP. Regarding kinetic profile, they reported that most transduction occurred within the first 30 min of perfusion and confirmed the existence of a dose-dependent response, with increased transduction rates with incremental titers infused.

## Discussion

The results summarized in this review highlight the feasibility and clinical potential of cardiac gene therapy during ESHP, especially considering the fact that both hypothermic (51) and normothermic (7, 8) ESHP have already been introduced in clinical practice. The evaluation of gene therapy in both temperature ranges is an interesting challenge by itself. Contrary to what might be expected, successful transduction could be achieved under hypothermic conditions, followed by expression of the inserted gene after heterotopic transplantation. This may be surprising, since hypothermia is associated with a significant reduction of enzymatic activity, reduced cellular respiration and metabolism (52), and, subsequently, reduced genetic processing of nucleic-acids used for genomic modification. Although the latter might be the result of subsequent rewarming (and hence restoration of metabolism with subsequent processing of the delivered genetic material) after transplantation, these result indicate that hypothermia does not have to limit vector viability or entry into cardiomyocytes and suggest that normothermic metabolism isn't a prerequisite for successful transduction. This is supported by the prolonged expression that was noted after gene therapy during hypothermic ESHP in multiples studies (36, 41–43). Furthermore, effective transduction during hypothermic ESHP might also be the result of the absence of components in the perfusate solution that negatively influence vector delivery, such as and plasma, since all studies use a crystalloid-based perfusate. Nonetheless, these results can be interpreted as evidence that the association between metabolism and efficacy of gene therapy might be more complex in nature than expected and also dependent on multiple other factors, like the specific vector used (53, 54) and composition of the perfusate solution (cellular vs. acellular).

Another important finding is that perfusion conditions, such as perfusion pressure and viral dose, seem to impact the efficiency of transduction in both hypothermic and normothermic conditions. Although heterogeneity regarding optimal vector dose is quite substantial, a pulsatile perfusion pressure around a mean of 50 mmHg seems required for optimal vector delivery and transduction. Endothelial integrity also plays an important role. Under normal physiological circumstances, the endothelium constitutes an uninterrupted barrier between the intravascular and extravascular compartment. When the endothelium becomes activated by pathological triggers, such as inflammation or excessively high pressures, gaps start to occur between the cells that facilitate diapedesis of leukocytes and contributes to edema formation. In the context of gene therapy, however, a certain degree of endothelial activation seems to be a prerequisite for success, as is evidenced by the finding that transduction efficiency during normothermic perfusion is improved by the addition of agents that increase microvascular permeability, a phenomenon that has also been previously observed for *in vivo* delivery studies (55). The harvesting of a donor heart is inherently associated with some degree of (warm) ischemia, even when ESHP is utilized. The subsequent reperfusion-associated injury will result in a varying degree of endothelial permeability (56, 57), which facilitates efficient gene transfer. This might hypothetically obviate the need for supplementation of agents that affect microvascular permeability in clinical situations that are associated with substantial (warm) ischemia, although this should be investigated in future studies. Increased microvascular permeability might also partially explain the observed transduction rates during hypothermia, as hypothermia in itself is associated with reversible morphological and functional changes to the endothelium that increase permeability (58). These findings are in accordance with what is known from studies that assess vector uptake in the myocardium during *in vivo* administration (17). However, given the fact that the above is investigated in preclinical models, one should be aware of a potential translational gap between small and large animal models and clinical efficacy in humans, as is evidenced by multiple clinical studies that failed to demonstrate efficacy in humans after convincing preclinical results in small and large animals (17, 20, 22). Future research, specifically designed to address these questions, seems a prerequisite before successful clinical implementation.

Central to the successful application of gene therapy, is efficiency of delivery (20). Efficiency of delivery in itself is dependent on the vector and the route of administration, both of which can be extensively controlled and manipulated during ESHP. Currently, potential vectors can be subdivided into viral and non-viral vectors. Non-viral vectors include liposomes, while extensive experimentation has indicated that two viral vector systems are effective at cardiac gene transfer, being Ad vectors and AAV vectors (13, 20). Generally speaking, Ad vectors have a high efficiency for delivery and expression of their genome within the target cells. They are effectively produced at high titers, are associated with rapid gene expression kinetics and can carry large genes due to their substantial insert capacity, in particular when considering the use of third generation, so-called “gutless” Ad vectors (59). However, important disadvantages are the significant associated immune response, triggering both innate and adaptive immunity which limits the duration of gene expression, and their innate tropism for many human tissues other than the heart, which can be circumvented during ESHP (17, 20). On the other hand, AAV vectors have the advantage that they are principally non-pathogenic and can have a high tropism for myocardium depending on serotype (60–62). They are also associated with sustained transgene expression. Disadvantages include their limited insert capacity, limiting the size of genes that can be transported, and relatively high production costs. They are also quite prone to evoking an immune response, antibody development and it seems that a large proportion of the general population seems to possess neutralizing antibodies for different AAV serotypes before treatment (63, 64), although this might be less of a problem during an ESHP approach. Based on the above, one can imagine that selection of the appropriate vector for delivery of the desired gene is not a straightforward process and depends upon many factors that influence outcome, including intention of treatment. Based on the available literature, Ad vectors currently seem the preferred vector for gene delivery during ESHP due to their large gene carrying capabilities, efficiency of transduction, the relatively low costs and favorable expression kinetics, which abrogates the theoretical advantages of AAV over Ad in the setting of ESHP. The higher immunogenic response that is associated with Ad therapy might also not be an issue during ESHP as it is possible to use a leukocyte-depleted perfusate, which may be supplemented with additional immunosuppressants (65), or use a perfusion approach that might not need any blood constituents, such as hypothermic ESHP. However, the limited long-term expression rate of genes delivered with Ad vectors might be an important barrier to long-lasting effects of gene therapy to treat pathogenic genetic mutations in carriers, unless they are used for gene-editing purposes (e.g., CRISPR-CAS). For gene delivery, however, this might be an important argument for the use of AAV over Ad in situations where a long-lasting expression of gene products is warranted, e.g., for replacing certain genetic defects or when long-lasting immunosuppression is desired (66), especially with the development of highly cardioselective serotypes, such as SASTG, that could greatly enhance efficiency of transduction (17, 20, 22, 67).

If implemented correctly, ESHP offers a unique opportunity for direct biological modification of a (defective) heart without the risks of systemic toxicity or side-effects. By isolating the organ in a metabolically and immunologically favorable condition, it is possible to directly investigate and manipulate the factors that influence important obstacles related to delivery and uptake that were encountered during *in vivo* clinical trials and potentially improve the success rate of cardiac gene therapy. By doing so, ESHP opens up the possibilities to improve quality of grafts, allow for selective immunomodulation to minimize the need for immunosuppression, improve tolerance to ischemia-reperfusion injury and further extend the donor pool. Ultimately, it could be used for correction of a genetic defect in known carriers of a pathogenic mutation after careful excision of the heart, preservation by machine perfusion and autotransplanting it into the same patient, which is supported by cardiopulmonary bypass during ESHP. By identifying and selectively treating these patients at an early stage, this could potentially ameliorate the need for heart transplantation altogether, thereby improving the current supply-demand mismatch and catalyzing a transition towards a new era of regenerative medicine and organ transplantation. Furthermore, lessons learned from experience with gene therapy during ESHP could also be employed in newly developed techniques for loco-regional isolated *in situ* heart perfusion, as previously described by White et al. (28) and which are currently being developed for clinical application (e.g., DiNAQOR AG (Schlieren, Switzerland). Especially in the light of recent developments in gene-editing techniques that might benefit from direct, isolated exposure to the target organ (e.g., CRISPR-CAS, TALENs) (68–70) and the recent successes in clinical application of gene therapy in other fields (71–74), these future prospects might be closer to reality than initially anticipated.

To summarize, key messages from the literature regarding the application of gene therapy during ESHP are that gene therapy is possible in both hypothermic and normothermic conditions, using Ad, AAV and liposomes. Perfusion conditions, such as pressure, duration of exposure to the vector, dose and perfusion composition seem to influence efficiency of transduction, while some degree of microvascular permeability is a prerequisite to successful application. To date, local immunomodulation and enhanced myocardial tolerance to ischemia-reperfusion injury have been achieved using gene transfer during ESHP in rodent models. Future studies should focus on replicating these findings in large animal models and humans, and the efficacy of gene therapy for the treatment of known mutations that affect heart function, such as mutations in phospholamban, lamin A/C, PKP2 or titin genes, using ESHP.
