# Supplementary material for: Gene therapy during ex situ heart perfusion: a new frontier in cardiac regenerative medicine?
Source: Front Cardiovasc Med. 2023 Oct 16;10:1264449. doi: 10.3389/fcvm.2023.1264449 (PMC10614057; doi:10.3389/fcvm.2023.1264449)
Supplement: Supplementary file 1 [file Datasheet1.docx]

**Supplementary files: search string**

Pubmed:

(((perfus*[Title/Abstract] OR reperfus*[Title/Abstract]) OR ("Perfusion"[Mesh])) AND ((heart*[Title/Abstract] OR cardiac[Title/Abstract]) OR ("Heart"[Mesh]))) AND (ex vivo[Title/Abstract] OR ex situ[Title/Abstract])

Embase:

((perfus*:ti,ab,kw OR reperfus*:ti,ab,kw) OR ('perfusion'/exp)) AND (heart*:ti,ab,kw OR cardiac:ti,ab,kw OR 'heart'/exp) AND (‘ex vivo’:ti,ab,kw OR ‘ex situ’:ti,ab,kw) AND [embase]/lim AND ('article'/it OR 'article in press'/it)
